# Online One-Stop Shop for Disaster Response Services After the MH17 Airplane Crash: An Evaluation Study

**DOI:** 10.3389/fpubh.2022.832840

**Published:** 2022-05-02

**Authors:** Merel M. van Herpen, Michel L. A. Dückers, Rick Schaap, Miranda Olff, Hans te Brake

**Affiliations:** ^1^ARQ Centre of Expertise for the Impact of Disasters and Crises, Diemen, Netherlands; ^2^Department of Psychiatry, Amsterdam Neuroscience & Public Health, Amsterdam UMC, Amsterdam, Netherlands; ^3^Netherlands Institute for Health Services Research (Nivel), Utrecht, Netherlands; ^4^Faculty of Behavioural and Social Sciences, University of Groningen, Groningen, Netherlands; ^5^ARQ National Psychotrauma Centre, Diemen, Netherlands

**Keywords:** one-stop shop, disaster response, psychosocial care, evaluation, online

## Abstract

**Background:**

A one-stop shop for disaster response services provides a central location for information and advice in an accessible way. Yet little is known about its organization and outcomes. After the MH17 airplane crash, the one-stop shop concept was realized through a digital environment called the Information and Referral Center (IRC). The aim of this study was to evaluate the experiences of users and providers in regard to the IRC and to identify improvement points for future IRCs.

**Method:**

Data was collected among affected ones as well as involved organizations, using interviews, focus groups, surveys and online user information. Existing evaluation and quality models were combined to design the study and analyze the data.

**Results:**

First, affected ones and a variety of organizations involved were positive about the merits of the IRC. Affected ones indicated they perceived the IRC as a reliable source of information and appreciated the referral possibilities. Second, the feature of the IRC to serve as a community where affected ones could meet, share experiences and support each other was hardly used according to participants. Lastly, tracking evolving psychosocial needs and problems through the IRC was hampered due to difficulty in accessing relevant data.

**Conclusions:**

The IRC helped organizations to structure and align their services. Affected ones were positive about its reliability and accessibility. An IRC has to be embedded within the established care structures. Future research could indicate whether an IRC is useful in other event types and population contexts as well.

## Introduction

On July 17, 2014, the disaster with the MH17 passenger flight from Amsterdam to Kuala Lumpur occurred above Ukraine. None of the 283 passengers and 15 crew members survived. Among the passengers were 196 individuals with the Dutch nationality. Consequently, the event had a severe impact on Dutch society among people directly and indirectly affected (1–6). The on-site investigation, recovering and identification of the bodies, and the criminal investigation were impactful moments, especially for people who lost a loved one. Wednesday July 23, 2014, was declared a day of national mourning in The Netherlands, and on November 10, 2014, an official commemoration took place. Exactly three years after the disaster, on July 17, 2017, a monument was revealed in memory of the deceased.

The day after the MH17 airplane crash, the Dutch government and organizations involved in the response decided to establish an online one-stop shop called the Information and Referral Center (IRC). The IRC was launched on July 18, 2014, and offered an online central location for information and advice regarding practical, legal and psychosocial matters. The aims of the IRC were to: (1) provide current, appropriate and reliable information and referral, (2) foster contact between affected ones, and (3) acquire information on needs, problems, and risk groups.

Based on experiences with earlier disasters since the 1990s, the one-stop shop has become a typical element of the psychosocial response to disasters and major incidents in The Netherlands (7). More broadly, the concept of a one-stop shop as a support structure for groups of affected people, fits logically within the international post-disaster psychosocial support knowledge base. Scholars have extensively documented the severe impact of disasters and crises, such as with the MH17 passenger flight, can have on the mental and physical health of affected individuals (8–16). There is broad consensus among experts about the importance of adequate post-disaster psychosocial service delivery (17–26). These services entail practical, legal and psychosocial support. Efficient coordination and integration of disaster response services should aid in the continuity of existing health care and provide psychosocial services to those affected by the disaster, which can be challenging due to chaotic circumstances and various demands (23).

In international guidelines of post-disaster psychosocial support the importance of providing affected individuals with a central coordination point or one-stop shop is emphasized (17–21, 27–31), especially in the first phase after a disaster (32). A one-stop shop integrates a variety of information and services in an accessible way. Yet little is known about its organization and outcomes. A one-stop shop can include several types of support, both online and physical. It can have an outreaching aspect by providing support and referral to professional care. At the same time, affected ones can turn to a one-stop shop for self-help. Local governments should be prepared to establish a one-stop shop to disseminate information (33) and coordinate the immediate response and long-term services in order to ensure service continuity (34). A needs assessment among municipalities in The Netherlands showed that respondents considered a one-stop shop as one location that affected ones can turn to for questions and help with practical, legal and health-related problems (35). Furthermore, it could serve as a solution to problems of psychosocial care after disasters that could be easily avoided (36). However, an evaluation of 40 post-disaster mental health and psychosocial support programs showed that less than half of the programs included an integrated coordination point for the long-term coordinated provision of psychosocial care services (33).

Evaluation studies are important because potential lessons from these studies can improve the provision of psychosocial support during future events (23). The importance of evaluating post-disaster interventions has been widely acknowledged in the literature (23, 24, 29, 37–43). At the same time, although crucial for learning purposes, research into the implementation of a program, “consumer access, uptake and outcomes” is modestly available in the international literature (23). To design the evaluation study and to structure and analyze the data, we used the evaluation framework by Stake (44, 45) that we combined with the healthcare quality model of Donabedian (46). The framework by Stake (44, 45) includes antecedents, transactions and outcomes. Stake (45) argues that outcome data usually receive most attention in evaluation studies, while the other two data sources are equally important. Donabedian (46) developed one of the most influential quality evaluation models applied to healthcare programs (38). This model distinguishes structure, process and outcome as quality categories and has been used before as an evaluation framework to assess the quality of multiple mental health and psychosocial support programs (37).

Both Donabedian (46) and Stake (44, 45) argue that it is essential to collect data from multiple sources to conduct a high-quality evaluation. In line with Donabedian (46), Stake (44, 45) argues that data on multiple domains should always be collected in order to draw conclusions about the quality of a program or intervention. All domains should receive equal attention instead of focusing mainly on the outcomes of a program. Stake's model (44, 45) is different from the framework of Donabedian (46) as it incorporates a comparison between the “intended” and “realized” program, while still recognizing the three interrelated components.

According to Stake (45) antecedents are various background conditions and inputs that can be indicators of quality. Data should be collected regarding the intentions, the actuality and the perceived quality of the program. E.g., collaboration between organizations while implementing a program. Antecedents relate to the quality information category “structure,” from the Donabedian model (46). Structure determines the context and conditions in which a program is intended and realized. It includes expectations about the program and the socioeconomic context as well. For example, the coordination within the provider network that determines the context.

Transactions are program activities, operations, functions and processes (45). E.g., a program aims to provide reliable information. This component relates to the quality information category “process” of Donabedian (46), relating to transactions between recipients and providers of care. In this study, we defined transactions as the methods of the instrument or intervention, in this case the IRC. This includes interaction with the target group; the affected ones.

Outcomes refer to data that provide insight in the accomplishments of the actual program. For example, providing psychosocial care that meets the needs of the recipient. According to Stake (45), a program will never be delivered exactly as intended because necessary changes have to be made along the way. The program in place should be evaluated and compared with the intended program. This can be linked to the quality information category “outcome” of Donabedian (46), that also refers to the actual outcomes of a program. Furthermore, Donabedian (46) emphasizes the importance of including the needs of the target group; which should be clear beforehand since the outcomes build on these needs.

The current study entails a systematic evaluation of the online one-stop shop service environment, planned and implemented after the MH17 disaster. To design the evaluation, we used existing evaluation and quality frameworks (44–46). Based on these frameworks, we examined the extent to which the antecedents (the structure and conditions that set the context), transactions (process; all activities and measures) and outcomes as envisioned, relate to the actual implementation of the IRC. Our objective was to evaluate the experiences of users and providers with the IRC using both qualitative and quantitative data and the evaluation frameworks by Stake (44, 45) and Donabedian (46). We aimed to answer the following research questions:

1. What were the experiences of users and providers in regard to the antecedents, transactions and outcomes of the IRC?
2. What were facilitating conditions and barriers in implementing the IRC and reaching its goals?
3. What potentially relevant implications for future IRCs can be identified?

## Materials and Methods

### Participants and Procedure

We collected data from three different sources: (1) affected ones, (2) online user information from the IRC website, and (3) employees from the organizations involved in the organization of the IRC, such as the Ministry of Justice and Security, the Ministry of Health, Welfare and Sports, the National Police, Public Prosecution Service, Victim Support Netherlands (Victim Support NL hereafter) and ARQ Center of Expertise for the Impact of Disasters and Crises (ARQ Impact hereafter).

ARQ Impact was head of the editorial council that was responsible for the IRC's development and implementation. The evaluation of the IRC was an integral part of its development. To ensure the independency of the evaluation and help develop the evaluation plan, a peer-review group consisting of four independent experts was established. The affiliations of the experts are included in the acknowledgments.

### Intended Program

**Figure 1:**
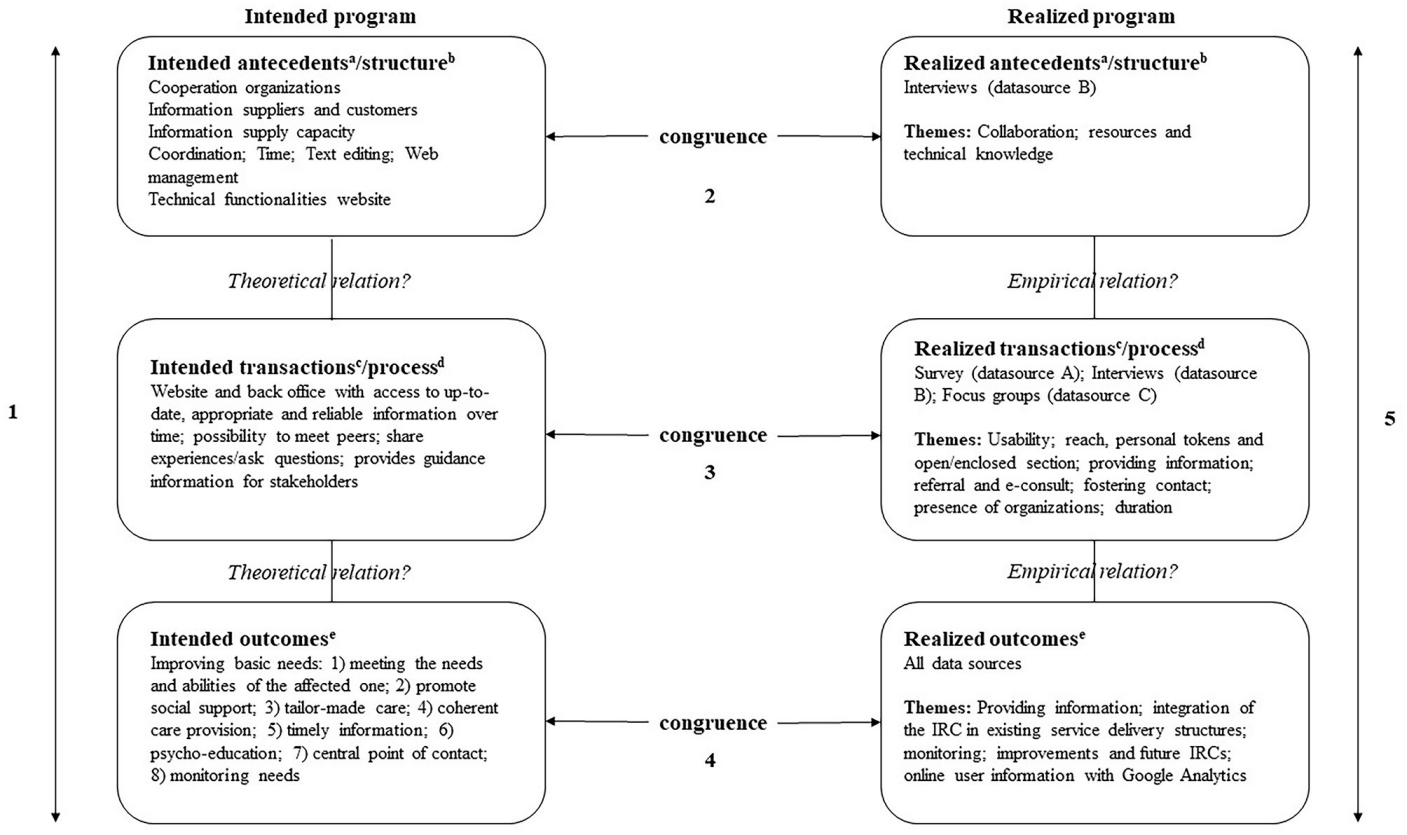
Evaluation framework of Stake (44, 45) and Donabedian model of quality (1988) applied to the IRC evaluation. ^a^Antecedents are various background conditions and inputs (45); ^b^Structure refers to the context and conditions (46); ^c^Transactions are program activities, operations, functions and processes (45); ^d^Process refers to transactions between care recipients and providers (46); ^e^Outcomes refer to the accomplishments or actual outcomes of the program (45, 46).

In this section, the intended program of the IRC is described, structured according to the evaluation framework (see the left side of Figure 1). The realized program is described in the Results (Paragraph Realized Program) based on the information from the three sources.

#### Intended Antecedents

Good collaboration between the organizations was essential for the successful development and implementation of the IRCs. Good collaboration is dependent on various conditions, such as trust, a central coordinator and conflict management. Dückers et al. (47) assessed the various organizations that play a role in the planning, implementation and evaluation of psychosocial care following disasters and crises in The Netherlands. A national collaborative of key organizations, consisting of governance organizations, coordinating organizations, executive organizations and a psychosocial care expert partner, was recommended. Depending on the various characteristics of a specific disaster or crisis, the collaborative has to be adapted.

An editorial council was established to facilitate the development and implementation of the IRCs content. The council consisted of Victim Support NL and ARQ Impact, together with representatives from the government, police and Public Prosecution Service. The collaboration within the editorial council was not formalized beforehand. Victim Support NL and ARQ Impact were responsible for the content and led the editorial council. The editorial council monitored and co-wrote new content for the IRC and ensured a consistency in style and form. Given the nature of the disaster, a group of experts regarding loss and traumatic grief were involved to advise the editorial council on grief and mourning. New content and the appropriate tone of voice were discussed by the council. In addition, Victim Support NL was responsible for the technical implementation, maintenance and development of new features of the IRC, in compliance with IT security guidelines. Another intended antecedent was the supply of information to IRC visitors. At the start of the IRC, all sections were accessible to all visitors.

#### Intended Transactions

The IRC was developed based on three main objectives. The first aim was to provide up to date, appropriate and reliable information and referral. In addition to people close to the deceased, the IRC was also established for other affected ones such as colleagues, but also institutions such as schools and leisure/sports organizations. The information was tailored to the different phases of the aftermath. If required, IRC visitors could be referred to (health)care providers. The second aim of the IRC was to foster contact between affected ones. The visitors were provided with the opportunity to contact each other through a forum on the enclosed section of the IRC. This also provided them with the opportunity to ask questions to other affected ones, public authorities and experts. The third aim of the IRC was to acquire information on needs, problems, and risk groups. The online environment generated group level information, which could help public authorities and providers of support services to decide if additional measures were required.

#### Intended Outcomes

The main intended outcome of the IRC was to improve psychosocial care for affected ones of the MH17 airplane crash. Based on the Dutch multidisciplinary guideline for psychosocial care after disasters and crises, psychosocial care could be implemented according to eight evaluation criteria. It should be: (1) an approach that starts from the needs and capacities of the affected one; (2) stimulating social support; (3) individually appropriate care, taking diversity among individuals affected into account; (4) offering care that is coherent and complementary, even though provided by different organizations; (5) providing incident-related information; (6) providing information on common emotional reactions; (7) providing a service point for questions and practical issues; and 8) monitoring individuals affected and initiating follow-up where needed (48).

### Data Sources and Measurements

#### Topic List

In this study, we collected data from different sources as recommended in the literature (44–46, 49). We developed a topic list based on the evaluation frameworks described above, input from the peer review group, and the aims of the IRC. See Table 1 and Figure 1 for an overview of the evaluation framework as applied to the evaluation of the IRC. The topic list guided all measures of this study. Not all topic list items were applicable to all measures and/or data sources.

**Table 1:** Topic list items and corresponding data sources and measurements.

	**Topic**	**Survey affected ones**	**Interviews involved organizations**	**Focus groups affected ones**
	**Antecedents**			
	***Collaboration between involved organizations***			
1	Collaboration in the beginning phase		X	
2	Current collaboration		X	
3	Possibilities to improve collaboration		X	
4	The formation of different teams, e.g., the editorial council and expert group		X	
5	A shared vision about the IRC		X	
6	Other organizations feeling actively involved		X	
7	Missing organizations from the collaborative		X	
8	Superfluous organizations within the collaborative		X	
9	Availability of sufficient budget		X	
10	Availability of sufficient technical knowledge		X	
11	Time pressure in the beginning phase and currently		X	
12	Availability of sufficient IT capacity		X	
13	The need for greater IT capacity investment for a future IRC		X	
	**Transactions**			
1	Usability: User friendliness	X	X	X
2	Usability: Clear structure	X	X	X
3	Usability: Lacking and superfluous features	X	X	X
4	Usability: Use of the forum			X
5	Usability: E-consult	X	X	X
6	Duration of the availability of the IRC	X	X	X
7	Security and accessibility	X	X	X
8	Personal token	X	X	X
9	Providing good psychosocial support with the IRC		X	
10	Coordinating the different organizations in providing psychosocial care in a coherent manner with the IRC			X
11	Fostering contact: Presence of other affected ones	X	X	X
12	Fostering contact: Sharing personal stories	X	X	X
13	Fostering contact: Opportunity to get in touch with others	X	X	X
14	Information: Reliability	X	X	X
15	Information: Meeting the needs	X	X	X
16	Information: Central location	X	X	X
17	Information: Comprehensibility	X	X	X
18	Information: Practical information, e.g., regarding mourning and loss	X	X	X
19	Taking into account the personal situation of affected ones and adapting accordingly	X	X	X
20	Referral to follow-up care		X	X
21	Presence of involved organizations on the IRC	X	X	X
22	Difference between open and enclosed section	X	X	X
23	Groups that were not reached		X	X
24	Ways to become familiar with the IRC	X	X	X
25	Moment of becoming familiar with the IRC	X	X	X
26	Visiting the IRC	X	X	X
27	Frequency of visits	X		X
	**Outcomes**			
1	The goal of the IRC	X	X	X
2	View on whether this goal has been reached		X	X
3	View on what the goal of the IRC should be		X	X
4	Potential improvements		X	X
5	Rationale for or against an IRC		X	X
6	Monitoring of affected ones		X	X
7	The complementarity and integration of the IRC to other available (care) resources			X

#### Survey With Affected Ones (Data Source A)

We developed a 14-item survey based on the topic list (see Table 1). All items are answered on a 5-point scale, ranging from 1 (completely disagree) to 5 (completely agree), also including answering option 6 (no opinion). To limit the burden on affected ones, the survey items were included in a larger study that was conducted by the University of Twente, University of Groningen, Intervict and ARQ Center'45. Written informed consent was obtained from all respondents (3). Affected ones were individuals who lost a loved one during the MH17 airplane crash, e.g., child, spouse, parent, sibling or other.

The survey was provided online. An invitation was posted on the enclosed section of the IRC in May 2015 and was included in the IRC newsletter as well. Furthermore, the Airplane Disaster MH17 Foundation (a representative body of affected ones) paid attention to the survey during general meetings and distributed the survey among affected ones. The survey was available until October 2015. Respondents who started the survey online but did not complete it received the survey hardcopy as well.

#### Interviews With Involved Organizations (Data Source B)

We conducted semi-structured interviews with participants employed by the different organizations that were involved in the development and implementation of the IRC. The interview guide was developed by the researchers. It was based on the evaluation framework and the study's overall topic list (see Table 1 and Figure 1) and focused on the perceptions of the different organizations regarding the processes of organizing the IRC. The interviews lasted between 1 and 2 hours and were conducted by two researchers (except for one interview). The interviews were conducted at the workplace of participants. Participants were representatives of their organization and the number of participants interviewed per organization was determined by the organization's responsibilities. Before the interview started, participants were informed of the goals of the study and the use of their information. Oral informed consent was obtained from all participants. This procedure was described in a manual, prior to the start of the study. At the end of the interview, participants were asked if any topics were not discussed. The interviews were recorded and the transcripts were sent back to the participants for approval, so aberrations could be corrected. Also, the transcripts were reviewed by the other researcher present at the interview.

#### Focus Groups With Affected Ones (Data Source C)

Focus groups were held with affected ones. Affected ones were individuals who lost a loved one during the MH17 airplane crash, e.g., child, spouse, parent, sibling or other. Participants were recruited through the survey [see Interviews With Involved Organizations (data source B)]. The topic list of the semi-structured focus groups was developed by the researchers, based on the evaluation framework and the study's overall topic list (see Table 1). During the focus groups, the use of the IRC was discussed, as well as improvements, information and resources.

Each focus group was chaired by a professional facilitator. The focus groups were conducted at two central locations in The Netherlands. The travel distance of the participants was taken into account. At least one researcher was present to make sure the topic list was fully covered. There was ample room for the expression of emotions and sharing personal stories, more open questions were asked. Therefore, the focus groups were less structured than the interviews (data source B). Because of the sensitive content and to provide participants with a maximum sense of safety, the focus groups were not audio recorded. Rather, elaborate minutes were made by the researcher that were transcribed elaborately afterwards. The minutes were reviewed by another researcher who was also present at the focus group. Before the interview started, participants were informed regarding the goals of the study and the use of their data. Oral informed consent was obtained from all participants. This procedure was described in a manual, prior to the start of the study.

#### Website Pop-Up Survey (Data Source D)

To evaluate the IRCs features and user friendliness, a brief pop-up survey was implemented on the IRC. Visitors were presented with the survey during their visit. The pop-up survey items are presented in **Table 3**. Items were answered on a 5-point scale, ranging from 1 (completely agree) to 5 (completely disagree). The pop-up survey was available from November 2015 until January 2016. No informed consent was obtained because no personal data was saved.

#### Google Analytics (Data Source E)

Google Analytics is a feature provided by Google and tracks website performance and collects data on visitor behavior. We used Google Analytics data to gain insight in trends of IRC user behavior. Unique page visits and information regarding a session were used to assess these trends. A session is defined as a set of user interactions that take place on a website within a certain period of time. For example, one session can include multiple screen or page views, events or social interactions.

### Analyses

The topic list and evaluation framework directed the analyses of the qualitative data from the interviews and focus groups and the analyses of the quantitative data from the survey and pop-up survey. The survey and pop-up survey data were analyzed using frequency distributions in SPSS. Google Analytics data was analyzed in Microsoft Excel.

Because our study is based on a theoretical framework and to be able to compare our samples, we took a deductive approach to analyze the qualitative data (50). A deductive approach helps focus the coding process on the evaluation framework by Stake (44, 45) and Donabedian (46) and assures structure and relevance to the framework. We developed the topic list based on the evaluation framework that guided all measures of the study and the coding process. In line with the deductive approach, we pre-defined a list of codes before data collection started (51). We ensured flexibility during the coding process so we would not miss new themes that emerged from the data. First, the interviews with the involved organizations (data source B) were reviewed and then coded based on the topic list in MAXQDA by RS. During the coding process, new themes were added when they emerged from the data. Authors RS and HtB discussed the codes of each separate interview and the themes until consensus was reached. Next, the focus groups with affected ones (data source C) were reviewed and then coded by RS. Again, authors RS and HtB discussed the codes of each separate interview and the themes until consensus was reached. During the coding process, we adapted the coding frame when new interesting themes emerged from the data that were related to the research questions or when we found that data was not coded by the existing codes. We compared the interviews and focus groups regarding similarities and differences between the themes. Next, the comparison and interpretation of the themes were discussed among all authors. To illustrate our findings and provide more insight into the experiences expressed by participants during the interviews and focus groups, quotes are presented throughout the Results between brackets.

## Results

### Participants

In total, 127 respondents completed the survey (data source A), 105 online. The sample was 57.5% female, 42.5% male. The mean age was 54 years old (SD = 15.5, range: 20–88 years). The level of education was rather high: 68.3% respondents completed higher education.

We interviewed 16 participants from 9 different organizations (data source B) between October 2015 and February 2016. The sample was 43.8% female. Furthermore, 22 affected ones participated in 5 focus groups (data source C) between July and October 2015. Six potential participants were recruited through the IRC, four participated. Via the survey, 43 potential participants were invited, of which 18 participated.

The pop-up survey (data source D) was completed by 25 respondents. The sample was 60% male and mean age was 61.6 years.

Google Analytics data was available from November 6, 2014 until February 15, 2016. The results showed 44.000 unique visits.

### Realized Program

In this section, the realized program of the IRC is analyzed according to the evaluation framework (see the right side of Figure 1). The corresponding data sources are specified.

#### Realized Antecedents

The supply of information to IRC visitors changed rapidly in the beginning of the IRC. At the start of the IRC, all sections were accessible to all visitors. After the first few days, on July 22, 2014, affected ones were provided with a special token to access an enclosed section of the website. This allowed for sharing information that was meant only for affected ones. In addition, it was possible to communicate on a private forum. Still some days later, from July 27, 2014, onwards, a newsletter was sent to all affected ones who received the special token. The newsletter summarized new information of the private section of the IRC.

##### Collaboration

The interviews with the involved organizations (data source B) were used to investigate the realized antecedents. Participants labeled collaboration in the starting phase as a “journey of discovery” *(“I think it was a bit messy, we were figuring out our activities, how to perform these, who should participate, deciding on the decision-making process, who had the final say, when to coordinate with each other and the different roles”* – Participant B1). Especially during the first weeks, the content and visual presentation of the IRC changed rapidly, as did the editorial council and the division of labor. Collaboration was perceived as complex due to group size, role unclarity and time pressure. Processes, division of labor and responsibilities within the editorial council were unclear and not formalized according to participants. However, participants regarded the collaboration as constructive *(“It was alright, we had to work together. We were able to launch the IRC very quickly and within a very short period of time”* – Participant B2). There was a shared perception of responsibility for developing the IRC, which made it easier to make decisions. Participants indicated that over time, the different tasks and responsibilities became more clear and the collaboration more efficient. For example, content and technical issues were discussed in separate meetings. Also, the number of organizations involved in the editorial board was decreased to facilitate more efficient collaboration.

Most participants indicated that one organization should be in charge of coordinating the content. Sufficient technical and human capacity was found essential. At first, Victim Support NL was responsible for posting all information on the enclosed section of the IRC. Due to a lack of capacity and because other organizations wanted to post their own information, this process was changed and other organizations could now post information as well. Not all participants agreed with this change, being afraid it would negatively affect the privacy and safety of the affected ones because of the visibility of their personal information on the enclosed section of the IRC *(“That is the moment I said, now we have to be very careful, it is not acceptable for affected ones to think that they are communicating with each other in a private environment, while in reality half the world is watching.”* – Participant B5). In addition, participants worried that it could lead to an inconsistent communication style.

Participants indicated that they thought the IRC's main objective was providing information and that this goal was clear to them *(“Providing information from all the different organizations involved and offering the possibility to affected ones to be able to ask questions to these organizations as well. That is the main feature of an application like this.”* – Participant B4). However, they felt a shared perspective on the content and implementation seemed to be lacking. The other goals of the IRC were unclear to participants. Participants of ARQ Impact indicated that the two other objectives of the IRC were not given enough attention. Participants indicated that the Dutch Ministry of Justice and Safety commissioned the IRC but did not formulate clear criteria. This allowed different interpretations.

During the interviews, participants indicated that providing information to affected ones did not always proceed correctly. It occurred that information was shared by the media or the IRC first, before the family detectives could communicate this to the affected ones. This was a problem for the family detectives as their relationship with affected ones could be damaged. Moreover, the police indicated feeling bypassed when Victim Support NL answered certain questions from affected ones without consulting the editorial council. Other participants felt that questions from affected ones remained unanswered for too long *(“Sometimes it would have been good if we had sent out a process statement as a response to questions that we didn't have an immediate answer to.”* – Participant B7).

Several participants from ARQ Impact, the police and the Public Prosecution Office felt they were not visible enough on the IRC because the branding style of Victim Support NL was used *(“We could be more visible as an organization in that respect. But when we were trying to achieve this, we got into a conflict and asked ourselves if that was worth it. But, in my opinion, next time it should be more clear that this is a joint effort and not just a Victim Support NL initiative.”* – Participant B7). Participants worried it was confusing to IRC visitors and it would lead to unclarity about the organization they were communicating with. Therefore, participants preferred a unique branding style for the IRC.

Most participants thought the collaborative was complete *(“No, I think we've been fairly complete, I wouldn't be able to say what other organizations should have participated.”* – Participant B3). However, a web developer could have been useful, because certain developments were not implemented due to technical limitations.

##### Resources and Technical Knowledge

Other antecedents assessed with the interviews with participants of involved organizations (data source B) were budget, time and technical knowledge. No budgetary limitations were experienced by participants. All participants mentioned the enormous time pressure in the acute phase after the crash as a point of concern *(“Yes, we were under time pressure. I know that this work is not for everyone. It is inherent in these types of situations (…) I thought we had two weeks to develop the enclosed section, but at a certain point it had to be finished the next day.”* – Participant B5). Almost all participants had to put (a lot of) their regular work on hold in order to be able to focus on the IRC.

All participants agreed that the IRC should be up to date and adapted to current technological standards. Participants described the IRC as adequate but outdated, static and limited *(“What I do struggle with sometimes is that the technical possibilities are quite limited. Which means a lot of things can't be done. I think that also restricts how appealing it can be made.”* – Participant B7). A dynamic website with possibilities to interact was not possible due to the outdated technology. Quick technical adjustments were limited because Victim Support NL outsourced the technical realization *(“I think there was limited capacity available to quickly tackle certain issues, resulting in having to wait for the next release, while an emergency release would have been more appropriate at certain times.”* – Participant B6). Functionalities such as a search engine, top-5 news items, and links to the newsletter became available at a later stage. Participants indicated that the IRC could not follow technical developments, including smartphone compatibility. Moreover, releasing new content was delayed due to limited capacity at Victim Support NL.

#### Realized Transactions

##### Usability of the IRC

Table 2A shows that 85.1% of affected ones perceived the IRC as valuable and 89.4% thought that its goals were clear (data source A). Respondents were less positive about the coordination of services and information provided tailored to personal situations, 30.8% viewed this in a negative light. In addition, not all respondents were satisfied with the user friendliness and structure of the IRC. This was in line with participants' experiences in the focus groups (data source C), during which participants stated that the IRC structure was a bit messy in the beginning *(“It looks messy, but I am able to find everything”* – Participant C1). This improved after a search engine was implemented. Nevertheless, participants indicated that a clear structure was lacking.

**Table 2:** Survey results of affected ones (data source A); frequency distribution in % *N* = 94.

**Statement**	**1**	**2**	**3**	**4**	**5**	**6**
The IRC^*^ has added value for affected ones	0	1.1	9.6	33.0	52.1	4.3
The IRC's^*^ objective is clear to me	0	3.2	6.4	46.8	42.6	1.1
The information is provided in an easy-to-understand language	0	2.1	3.2	63.8	29.8	1.1
Questions of affected ones to involved organizations are taken seriously	0	1.1	14.9	48.9	29.8	5.3
The personal token performs well	2.1	4.3	7.4	42.6	41.5	2.1
The IRC^*^ is easy to use	3.2	12.9	14.0	46.2	20.4	3.2
The IRC^*^ is clearly structured, I can find the information I am looking for	5.4	17.2	17.2	40.9	18.3	1.1
Services and information provided by the IRC^*^ could be better tailored to my personal situation (e.g., age)	2.1	28.7	39.4	11.7	6.4	11.7

*Range: 1 (completely disagree), 2 (disagree), 3 (neutral), 4 (agree), 5 (completely agree), 6 (no opinion);*

* *Information and Referral Center; IRC*.

In general, the focus groups with affected ones (data source C) showed that participants were satisfied with the IRC. Especially in the first months, participants noticed the developments of the IRC that they assessed as improvements *(“Every change was an improvement. The IRC became more complete. If I was missing something, I would ask questions and those were addressed.”* – Participant C2). In addition, being able to ask questions to organizations was appreciated. However, some participants perceived not all questions were answered quickly.

Predominantly, participants (data source C) appreciated that information was first shared with them before it was published by the media. The information was perceived as reliable and it met their needs. Moreover, it followed new developments, which was appreciated *(“It feels like a digital companion. Warm and familiar. That is because of the quick responses, being able to find things, and that it is being updated.”* – Participant C3). Shortly after the crash, information was predominantly of practical nature. Later on, more attention was given to topics like grief and peer support.

Although the IRC consisted of an enclosed section, information leaked to the media nonetheless. Participants (data source C) thought this was difficult to prevent. Fellow affected ones were responsible for sharing the information *(“Keeping it private is impossible. Because people can pass on their tokens. That's hard to prevent.”* – Participant C4). Participants did not think of this as a priority and said not everyones needs can be met considering the large group of affected ones.

Overall, participants of the involved organizations (data source B) were satisfied with the IRC performance, especially given the time pressure and the difficult task to provide affected ones with information from the different organizations. The primary feature of the IRC, providing information, was developed accordingly and was experienced as effective *(“I think the IRC is a very good addition to the source of information toward the affected ones, an information repository. Also, the possibility to communicate in a private environment given the public and enclosed sections is beneficial.”* – Participant B10).

##### IRC Reach, Personal Tokens and Open/Enclosed Section

Data source A provided more information about IRC use and reach, see Tables 2B–E for details. The survey results show that 90% of affected ones were informed about the IRC, mostly by a family detective. 91.8% of the respondents were informed within one month after the plane crash. The survey results were in line with the results of the focus groups (data source C). Most participants when informed about the IRC, visited the IRC directly. Others indicated they did not desire to visit the IRC right away and did so at a later moment. Most participants had no clear expectations when first visiting the IRC but hoped to find information and to ask questions *(“Information. The news was filled with: “probably”. I only accept facts from the government. I used the IRC to fact-check information.”* – Participant C5). Some participants also expected interaction between affected ones.

**Table 2B-E:** Survey results of affected ones (data source A).

	**% (*N*)**
2B. How were you informed regarding the establishment of the IRC^*^?	
Family detective	46.0 (58)
Information meeting Nieuwegein July 21, 2014	16.7 (21)
I was not informed	10.3 (13)
Through other affected ones	9.5 (12)
Other	9.5 (12)
Case manager of Victim Support Netherlands	7.9 (10
Total	100 (127)
2C. When were you informed regarding the establishment of the IRC^*^?	
Within a week after the plane crash	42.7 (47)
Between a week and a month after the plane crash	49.1 (54)
More than a month after the plane crash	8.2 (9)
Total	100 (110)
2D. Did you visit the IRC^*^?	
Yes (at least once)	84.1 (95)
No, I did not visit the IRC, because…	15.9 (18)
Total	100 (113)
**2E. Did you use the personal token?**	
Yes, I used my token	90.5 (86)
No, I did not receive a token	1.1 (1)
No, my family's contact person uses the token and informs us	5.3 (5)
No, I did not use the token, because…	3.2 (3)
Total	100 (95)

* *Information and Referral Center; IRC*.

Most respondents, 84.1% (data source A), visited the IRC at least once. Respondents who did not visit the IRC indicated they received information from others and did not need the IRC. Most respondents visited the IRC on a daily basis. Visitation numbers decreased over time from daily visits to weekly. Most survey respondents (90.5%) indicated they used their personal token to access the enclosed section of the IRC. The difference between the open and enclosed section was not clear to many affected ones (data source C). Most of them said they only visited the enclosed section. They also thought the IRC was only meant for affected ones while the publicly available information could be useful to others, such as friends, as well. The enclosed section was perceived as safe *(“It gave a nice feeling of being in a protected environment.”* – Participant C11). The difference between the open and enclosed section was also not clear to all participants from the involved organizations (data source B). The involved organizations (data source B) considered the safety of the enclosed section of the IRC as sufficient *(“All of the security issues have been resolved at a very high pace, so overall, I'm very happy with it.”* – Participant B5).

Affected ones (data source C) indicated during the focus groups that they visited the IRC on a daily basis in the beginning, sometimes multiple times a day *(“Very often, every day, visits sometimes lasted up to an hour and a half. Due to the changing flow of information I felt the urge not to miss a thing.”* – Participant C6). Their visits decreased over time, partially because other information channels were established and partially because the amount of new information itself decreased. Participants indicated that they used the IRC newsletter (sent by Victim Support NL) to determine what messages they preferred to read. Visitation time varied greatly between participants, from 10 to 90 minutes.

##### Providing Information

Table 2F shows that 90.4% of affected ones (data source A) considered the information provided on the IRC as reliable. In addition, most respondents (95.8%; data source A) deemed it important that information was posted on the IRC first before it was published in the media. Affected ones indicated during focus groups (data source C) that they predominantly searched for information from the government or service providers. Mainly in the early stage of the IRC the need for information was high *(“Information, all kinds of information, we wanted to know everything.”* – Participant C7).

**Table 2F:** Survey results of affected ones (data source A), frequency distribution in % *N* = 94.

**Statement**	**1**	**2**	**3**	**4**	**5**	**6**
It is important the information is posted on the IRC^*^ before it is published by the media	0	0	2.1	16.0	79.8	2.1
It is important the IRC^*^ provides information in one central location	1.1	0	4.3	24.5	69.1	1.1
The information on the IRC^*^ is reliable	0	0	8.5	40.4	50.0	1.1
The information meets my needs	2.1	0	19.1	48.9	28.7	1.1
The information is clear and complete	0	3.2	19.1	48.9	26.6	2.1

*Range: 1 (completely disagree), 2 (disagree), 3 (neutral), 4 (agree), 5 (completely agree), 6 (no opinion);*

* *Information and Referral Center; IRC*.

Participants of the involved organizations (data source B) were satisfied with the information feature of the IRC. All participants stated the importance of providing reliable and up to date information to affected ones in one central location. Participants also indicated the importance of background information—such as legal and practical information—and information on grief and mourning. Participants considered the coordination between the organizations involved in providing information to affected ones as a great strength of the IRC *(“People in these kinds of situations just want sound and high-quality information. The information provided has to be backed up by all organizations involved, that is a great strength of the IRC.”* – Participant B10).

Participants (data source B) stated that the way in which information was provided could be improved by introducing more variation *(“There could be more, a bit more interactive and with more energy. Recently, we have been making videos that still need to be posted. We have been making blogs that also need to be posted.”* – Participant B2). Affected ones may have difficulties with concentrating, therefore long text may not be appropriate and the structure could be improved.

The IRC also served as an archive. This provides the opportunity to retrieve and read information previously posted on the IRC. Given the continuous supply of information, the archive was considered important to affected ones to allow them to read the information at a later stage. Retrieving archived information was perceived as convenient and was appreciated by affected ones (data source C).

##### Referral and e-Consult

Participants of the involved organizations (data source B) stated it could be useful to affected ones to be able to screen themselves for (mental) health problems *(“Perhaps adding types of e-health tools to assess how you are doing, based on ten questions to see how you are feeling or whether you should seek help in case of a certain outcome.”* – Participant B9). Additionally, available care should be outlined in a clear manner. Most participants indicated they had limited insight in the performance of the referral feature.

Table 2G shows that 72.7% of the survey respondents (data source A) were aware of and positive about the e-consult feature. However, respondents were less positive about using the e-consult or recommending it to someone else. Not all participants of the focus groups (data source C) were aware of the existence of the e-consult. Most participants stated they would not use the e-consult because they preferred their own resources *(“I have read it, but have already found my own way. Otherwise, I can also talk to the case manager of Victim Support NL. I already had my own resource for questions.”* – Participant C8).

**Table 2G:** Survey results of affected ones (data source A), frequency distribution in %, *N* = 95.

**Statement**	**1**	**2**	**3**	**4**	**5**	**6**
The e-consult is beneficial	0	0	18.9	43.2	29.5	8.4
I would recommend the e-consult feature to someone that has questions regarding mourning and loss or when I want to know where one can go for psychological help	2.1	7.4	23.2	30.5	25.3	11.6
I would use the e-consult feature when I have questions regarding mourning and loss or when I want to know where I can go for psychological help	5.3	16.8	22.1	28.4	20.0	7.4

*Range: 1 (completely disagree), 2 (disagree), 3 (neutral), 4 (agree), 5 (completely agree), 6 (no opinion)*.

##### Fostering Contact Between Affected Ones

The IRC feature of communicating with other affected ones was used very little. Participants of the involved organizations (data source B) indicated that affected ones met each other in person instead *(“I think there are few calamities imaginable whereafter so many meetings were organized in such a short period of time. So all things considered (…) we have had at least 5 information meetings.”* – Participant B5). Also differences in background, stage of the mourning process and needs in having contact with other affected ones influenced this.

Table 2H shows that a little over half of respondents (59%; data source A) agreed the IRC should offer the possibility of getting in touch with other affected ones. Respondents were negative about feeling supported by other affected ones (17.9%; data source A). Table 2I shows that 68.4% did not wish to get in touch with other affected ones through the IRC. Only 16.8% met others through the IRC. 14.7% wanted to get in touch but did not manage to do so yet.

**Table 2H:** Survey results of affected ones (data source A), frequency distribution in %, *N* = 95.

**Statement**	**1**	**2**	**3**	**4**	**5**	**6**
I think that the IRC^*^ should provide the opportunity to get in touch with other affected ones	0	3.2	30.5	37.9	21.1	7.4
The presence of other affected ones on the IRC^*^ makes me feel supported	5.3	12.6	27.4	36.8	13.7	4.2
Because of the IRC^*^ I feel like I am in touch with other affected ones	9.5	14.7	31.6	26.3	11.6	6.3
I think it is important that other affected ones can respond to my story	10.5	12.6	41.1	17.9	9.5	8.4
I think it is important to share my story on the IRC^*^	13.7	15.8	41.1	15.8	7.4	6.3

*Range: 1 (completely disagree), 2 (disagree), 3 (neutral), 4 (agree), 5 (completely agree), 6 (no opinion);*

* *Information and Referral Center; IRC*.

**Table 2I:** Survey results of affected ones (data source A), *N* = 95.

Have you made contact with other affected ones through the IRC^*^?	**% (*N*)**
No, I did not want to	68.4 (65)
Yes	16.8 (16)
No, I want to but did not manage yet	14.7 (14)
Total	100 (95)

* *Information and Referral Center; IRC*.

The results of the focus groups with affected ones (data source C) showed that participants perceived the forum as a useful addition. The need for sharing personal stories varied among participants. This was due to individual needs and also with the forum's atmosphere, that was determined by a small group of visitors who posted frequently. Participants indicated they preferred face-to-face contact instead of the forum. Other participants shared positive experiences with sharing their story on the forum (“*I asked a question once. It made a safe impression, not that I was exposing myself in front of the whole world.”* – Participant C9).

The experiences of participants (data source C) in getting in touch with other affected ones varied. Some had a positive experience while others expressed they thought the IRC was not the appropriate location for peer contact, because it was too large scale *(“When people communicate with each other through the IRC it creates chaos. The group is too large and too diverse. Therefore, I think you should not be looking for that part on the IRC.”* – Participant C10). Other participants had no desire for getting in touch with other affected ones at all.

##### Presence of Involved Organizations

The focus groups with affected ones (data source C) showed that it was not clear to all participants what organizations were involved in the IRC and what their different responsibilities were. Participants appreciated the presence of the involved organizations in one central location (“*It was nice that everything was posted in one location.”* – Participant C12). They were positive about the information the government posted on the IRC and the possibility to ask questions.

##### Duration of the IRC

The intended duration of the IRC was 2 years. All participants of the involved organizations (data source B) considered this as the minimum. More than half of affected ones (60.6%; data source A) agreed with this. See Table 2J for details. Participants (data source B) differed in opinion whether the IRC should be available for a longer period of time *(“The functionalities of the open section should not be deleted, perhaps it could be archived. But the enclosed part of the IRC should be scaled down, two years is an appropriate amount of time for it to continue”* – Participant B8). Features such as the information archive and e-consult could remain active after 2 years. Most participants of the focus groups (data source C) stated that the IRC should be available for a longer period of time *(“To me, it's not over until the perpetrators are in jail. Until then you want to have a location where all that information is stored.”* – Participant C13). Ending the IRC should be communicated clearly and proceed slowly. Participants wished to maintain the information archive feature of the IRC.

**Table 2J:** Survey results of affected ones (data source A).

For how long should the IRC^*^remain available to you?	**% (*N*)**
The IRC can be canceled now	3.2 (3)
At least 1 year after the event	21.3 (20)
At least 2 years after the event	38.3 (36)
More than 2 years after the event	22.3 (21)
No opinion	14.9 (14)
Total	100 (94)

* *Information and Referral Center; IRC*.

#### Realized Outcomes

##### Primary Goal of the IRC

Participants of involved organizations (data source B) indicated that providing reliable information in one central location as the primary goal of the IRC, before it was published by the media. Participants stated that this objective has been achieved *(“In my opinion, you can really find everything you need there.”* – Participant B5). Affected ones (data source C) also considered providing information as the main goal of the IRC *(“Reliable information, that was good.”* – Participant C14).

Most respondents of the pop-up survey (80%; data source D) thought the information of the IRC was easy to understand. 36% of respondents (data source D) were negative about connecting with (the experiences of) others through the IRC. See Table 3 for details.

**Table 3:** IRC^*^ pop-up survey results (data source D), frequency distribution in %, *N* = 25.

**Statement**	**1**	**2**	**3**	**4**	**5**
I consider the IRC^*^ easy to use	8.0	8.0	28.0	32.0	24.0
The information on the IRC^*^ is easy to understand	8.0	0	12.0	60.0	20.0
I can easily find the information I am looking for on the IRC^*^	8.0	12.0	20.0	48.0	12.0
The IRC^*^ has helped me to connect with (the experiences of) other affected ones	16.0	20.0	32.0	20.0	12.0
The information on grief and loss has been very helpful to me	8.0	16.0	24.0	32.0	20.0
I have benefited a lot from the practical and legal information	8.0	12.0	20.0	44.0	16.0
I have benefited greatly from the information provided by the organizations involved	8.0	4.0	28.0	32.0	28.0
I appreciate the opportunity to respond to messages from the organizations involved	4.0	4.0	20.0	48.0	24.0
The IRC^*^ has been an important part of the psychosocial care provided to me	12.0	8.0	16.0	32.0	32.0

*Range: 1 (completely disagree), 2 (disagree), 3 (neutral), 4 (agree), 5 (completely agree);*

* *Information and Referral Center; IRC*.

##### Integration of the IRC in Existing Service Delivery Structures

All participants (data source B) agreed that the IRC should complement existing healthcare and support facilities *(“Also, when it comes to referral, the IRC is complementary. The e-consult is not the gateway to all care, it's meant for the people who don't get to the right place through regular routes. I don't think the IRC has a main role in everything but that it is complementary.”* – Participant B7). These have been outlined and information on how to find support are provided on the IRC. In addition, the IRC can refer visitors to care through the e-consult. Participants (data source B) indicated they had limited insight in the performance of this feature. Affected ones (data source C) expressed a need for contact with fellow affected ones. The IRC could be an appropriate tool for this *(“Yes, it would have been nice to have some contact with peers, on a forum. That would have brought recognition and acknowledgment (…) I would like to get in touch with others who have a similar relationship with the deceased one, so you are able to share the same dynamics that are at play.”* – Participant C15). Still, participants expressed reluctancy in getting in contact through the IRC. They indicated this was partly due to previous experiences with the IRC.

##### Monitoring

With regard to the monitoring of affected ones, some participants (data source B) indicated that they had expected to get a clearer picture of the (mourning) process of affected ones and their associated needs *(“I expected that we would get a clearer picture of where people are, what their needs are. We haven't really been able to do that now. We don't really have a tool for that now.”* – Participant B7). Only few participants identified the peer support feature of the IRC as important. Those who mentioned peer support, indicated that they saw it as a subordinate feature. Participants indicated that this goal of the IRC did not receive enough priority due to a lack of time and capacity.

##### Improvements and Future IRCs

Participants (data source B) expressed that technical capacity is essential to meet user expectations in future IRCs. Participants explained that if what is provided does not match user expectations, it could reduce the effectiveness of an IRC. To anticipate this, most participants (data source B) proposed realizing a “basic IRC” that receives frequent maintenance *(“A sort of annual drill to assess if it all still works, if we know what we are doing, and how everything works.”* – Participant B9). Participants were aware of the required financial and material resources as complicating factors.

Respondents of all organizations (data source B) indicated the importance of sustaining the IRC collaborative for a rapid collaboration during future events (“*To keep direct lines of communication so that when the time comes we know how to find each other.”* – Participant B11). They indicated that this collaborative should include at least the government, Victim Support NL, ARQ Impact and a website developer.

In regard to potential improvements, several affected ones (data source C) suggested during the focus groups that the tone of voice on the forum could be monitored by a moderator *(“I do recognize that you shy away from that anger that people showed. You actually need to get a moderator on that.”* – Participant C16). Giving a moderator such a role should be implemented with caution. Also, language was perceived as too complicated and texts too long by some participants. This could be improved by providing summaries.

##### Online User Information (Data Source E)

**Figure 2:**
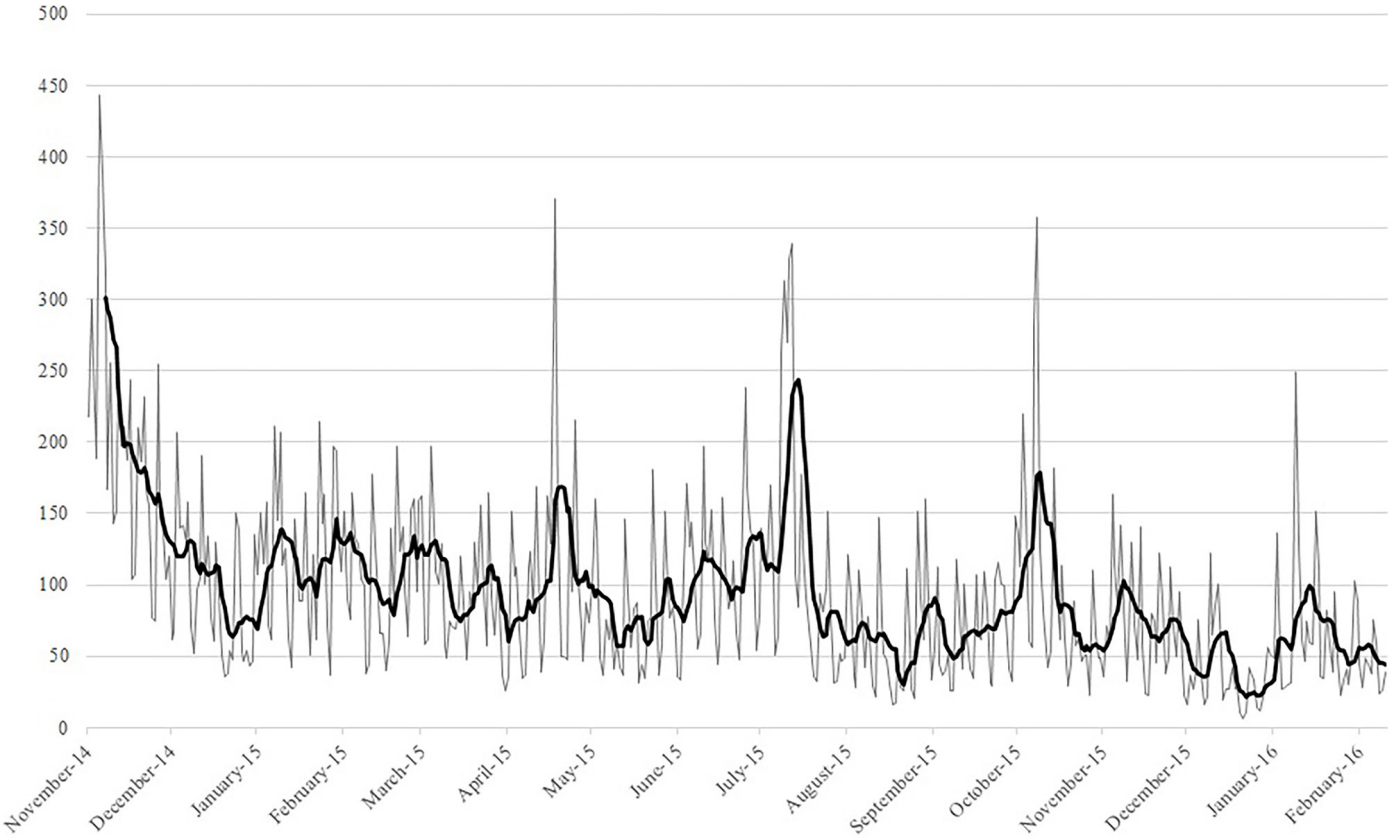
IRC daily sessions from November 6, 2014, until February 15, 2016.

Google Analytics data was available from November 6, 2014, until February 15, 2016. Due to technical issues, data from July 2014 until October 2014 was not stored and therefore unavailable for analysis. The results are presented in Table 4. The results showed over 44.000 sessions from 1 IP address. Most visitors were from The Netherlands. Figure 2 presents the number of sessions from November 6, 2014, until February 6, 2016, that shows a slight decline. The number of weekly visitors from early 2015 to February 2016 declined from approximately 150 to between 50 and 100 visits. In addition, a fluctuation in the number of sessions is shown as well. Visitation peaks are in concurrence with specific events or moments involving increased (media) attention, see Table 5.

**Table 4:** IRC^*^ Google Analytics data (Data source E) from November 6, 2014, until February 15, 2016.

**Google Analytics**	
Number of sessions	44.429
Total number of visited pages	366.108
Average page views per session	8.42 pages
Average session time	4 min 16 s
Dutch	93.9%
Foreign	6.1%

* *Information and Referral Center; IRC*.

**Table 5:** IRC^*^ Google Analytics data (Data source E) visitation peaks.

**Date**	**Subject**	**Numbers**	**Peak**
April 22, 2015	Sensitive information was shared in a Dutch TV news show for study purposes, that became public and led to public commotion.	343	Yes, additional IRC^*^ newsletter sent
April 30, 2015	End of repatriation mission	184	Yes, additional IRC^*^ newsletter sent on this date
May 1, 2015	Debate in House of Representatives (Minsk II)	74	No
June 15, 2015	Letter LTFO^**^ about professor Maat	189	Yes, additional IRC^*^ newsletter sent on this date

* *Information and Referral Center; IRC*.

** *Dutch National Forensic Investigation Team (LTFO)*.

Google Analytics is a useful tool to monitor user behavior. Unfortunately it has not been used to its full potential in case of the IRC because reliable data from the beginning of the IRC (before November 6, 2014) is missing. Also, no data was tracked regarding the use of the personal tokens or visitation differences on the open and enclosed sections. Nevertheless, the data that was gathered provided a useful insight in user behavior trends.

## Discussion

The objective of this study was to evaluate the experiences of users and providers with the online Information and Referral Center (IRC) established after the MH17 airplane crash using both qualitative and quantitative data and the evaluation framework based on Stake (44, 45) and Donabedian (46). First, regarding the experiences of users and providers in regard to the antecedents, transactions and outcomes of the IRC (research question 1); participants were positive about the merits of the IRC. Affected ones indicated that they perceived the IRC as a reliable source of information and appreciated the referral possibilities. Organizations stated that the IRC helped them to structure and align their services. The feature of the IRC to serve as a community where affected ones could meet, share experiences and support each other was hardly used according to participants. Tracking evolving psychosocial needs and problems through the IRC was hampered due to difficulty in accessing relevant data. Second, several facilitating conditions and barriers in the implementation of the IRC could be identified (research question 2), such as good collaboration, having sufficient resources and technical capacity, and the diversity of needs that changed over time. Third, potential relevant implications for future IRCs (research question 3) from the point of view of affected ones included clear communication regarding the functionalities and goals of an IRC. From the organizations' point of view, potential relevant implications regarded role clarity, a shared vision regarding goals and functionalities, and clear agreements beforehand.

As can be expected in the aftermath of a disaster or crisis, the intended program was not worked out meticulously beforehand. In addition, the intended and realized program were not entirely congruent because necessary changes were made along the way. The evaluation framework as used in this study includes an interpretation of the congruence between the intended and realized program, see Figure 1. First, the interpretation of the congruence between the intended and realized antecedents concerned the collaboration between the organizations involved in the development and implementation of the IRC. This collaborative concerned an opportunistic structure of organizations in which key players actively engaged. Because the process, division of labor and responsibilities were not formalized beforehand, resulting in role unclarity that complicated the collaboration. The process of who could post information on the IRC was changed along the way. Also, a joint perspective on the content and implementation was lacking and the second and third goal were unclear to some organizations and were given insufficient attention.

Second, the interpretation of the congruence between the intended and realized transactions concerned the background conditions, program activities and goals of the IRC. The structure of the IRC was perceived as messy in the beginning by users, but this improved along the way. Features such as a search engine and archive function were implemented at a later stage, improving the intended program according to users.

Third, the interpretation of the congruence between the intended and realized outcomes relate to the implementation of the IRC according to eight evaluation criteria (48). First, the IRC focused on the needs and capacities of the affected ones (principle 1) by adapting to the different phases of the aftermath. Through the IRC, organizations tried to encourage social support (principle 2) through the enclosed section and forum option, that did not work as expected. Because the group of affected ones was so diverse, it sometimes proved difficult to take all the different needs into account (principle 3). Affected ones were provided with the opportunity to ask questions to the different organizations involved and a referral option was established, thereby offering coherent and complementary care (principle 4). The information posted on the IRC followed incident-related developments (principle 5) and information regarding common emotional reactions, grief and mourning was provided (principle 6). The IRC also provided information on practical and legal matters, and visitors could ask questions (principle 7). The monitoring of affected ones and initiating follow-up where needed (principle 8) was difficult due to limited user data.

Our study suggests that the IRC had value in the aftermath of the MH17 airplane crash. According to users, the IRC delivered what affected ones perceived as important; reliable information and referral options. It indicated that an IRC has the potential to serve as a valuable psychosocial care instrument, both in the acute phase of a disaster (32) and in the longer term. Furthermore, it helped organizations to structure and align their services (34). Our study provides a comprehensive and in-depth understanding of the experiences of both users and providers of an intervention such as the IRC.

To our knowledge, this is one of the first evaluations of a post-disaster intervention such as the one-stop shop using both quantitative and qualitative data and including users and providers. By including multiple data sources, we did not only focus on the outcomes of the IRC, but also on the background conditions and program activities. Although the inclusion of data from affected ones is a strength of this study, our sample is a convenience sample which results in limited generalizability. Given the nature of the event, retrieving a representative sample was difficult while also limiting the burden on the population. In addition, the small sample size of the survey and pop-up survey increases the risk of selection bias, this should be kept in mind when interpreting the results. Another limitation of our study is that the qualitative data—the transcripts from interviews and focus groups—were coded by one researcher only, where this ideally is done by multiple researchers. However, the codes and interpretations were discussed extensively among a team of researchers. Including multiple researchers (triangulation) in data collection and analysis decreases the risk of bias. Furthermore, limited availability of Google Analytics data restricts drawing conclusions regarding user behavior. Lastly, each disaster and its aftermath is unique, this is also the case for the MH17 airplane crash. This study focuses on this particular event and therefore caution should be exercised in generalizing the results to other settings. This includes taking into account that the availability of resources will vary between countries and this IRC was implemented in a country with ample resources available.

From the perspective of affected ones, a potentially relevant implication for future IRCs is that clear communication regarding the functionalities and goals of an IRC is required. For example, the open section of the IRC contained of a lot of information that was publicly available, while this was not clear to everyone. Most affected ones and organizations indicated that they focused on the enclosed section. The services of the open section could have been communicated by national and regional media outlets. Website design could aid in this as well. Moreover, affected ones stated that they appreciated receiving information through the IRC before it was published by the media; this is an aspect of the IRC that should definitely be implemented in future IRCs as well. Needs regarding the IRC varied between individuals and also changed over time, as is typically the case with disasters. An IRC that monitors these changing needs and adapts accordingly fulfills an invaluable psychosocial crisis management function (52). The archive feature of the IRC, which can help in the mourning process, should be implemented. The atmosphere on the forum was perceived as unsafe by some affected ones. Therefore, a private chat feature could be considered for future IRC.

What we can learn from the perspective of the involved organizations is that role clarity is essential to successful implementation. A shared vision on the goals and functionalities of the one-stop shop support environment is important according to participants. Collaboration would benefit from clear agreements beforehand. This is difficult to realize in the acute phase following a disaster and something that probably needs to grow as the platform and the network behind it evolves. Nevertheless, attention should be paid to selecting the organizations that have to be included in the collaborative at an early stage. It is important to develop a scenario with clear roles and responsibilities, that can guide future IRCs. Participants recommended preparing and developing information for future service delivery platforms as far possible, so this is readily available during future crises. Participants proposed to develop and maintain a basic IRC for training purposes and as a starting point for acute situations. All this could enable and structure prompt collaboration at the time of a new disaster. This corresponds with a study by Bonfield (33), that suggests that governments should be ready to implement an one-stop shop. Furthermore, user friendliness according to current modern standards and cyber security should receive ample attention according to participants. Lastly, including sufficient technical capacity in the collaborative and ensuring cyber security was also deemed important.

In conclusion, this study suggests that an IRC has the potential to be a useful and appreciated psychosocial support instrument that, in the case of the MH17 airplane disaster, helped organizations with aligning their communication and interactions after the MH17 airplane crash, internally as well as toward target groups. Affected ones were positive about the IRC, predominantly about its reliability and accessibility. Like any psychosocial intervention, an instrument such as an IRC has to be embedded within the established structure of care providers. Future research could indicate if an IRC is useful in other event types and population contexts as well, and indicate what aspects of an IRC are deemed most important by users and providers.

## Data Availability Statement

The datasets presented in this article are not readily available because of the privacy of participants. Requests to access the datasets should be directed to MH; m.van.herpen@impact.arq.org.

## Ethics Statement

Ethical review and approval was not required for the study on human participants in accordance with the local legislation and institutional requirements. Written informed consent for participation was not required for this study in accordance with the national legislation and the institutional requirements.

## Author Contributions

RS, HB, and MD contributed to conception and design of the study and conducted the initial analyses. RS and HB collected the data. MH wrote the first draft of the manuscript. All authors contributed to the analyses and interpretation of the results, contributed to manuscript revision, read, and approved the submitted version.

## Funding

This study was funded by the Dutch Ministry of Justice and Security. The Ministry had no involvement in the writing of the manuscript.

## Conflict of Interest

The authors declare that the research was conducted in the absence of any commercial or financial relationships that could be construed as a potential conflict of interest.

## Publisher's Note

All claims expressed in this article are solely those of the authors and do not necessarily represent those of their affiliated organizations, or those of the publisher, the editors and the reviewers. Any product that may be evaluated in this article, or claim that may be made by its manufacturer, is not guaranteed or endorsed by the publisher.
